# Enhanced Proton Conductivity in Sulfonated Poly(ether ether ketone) Membranes by Incorporating Sodium Dodecyl Benzene Sulfonate

**DOI:** 10.3390/polym11020203

**Published:** 2019-01-24

**Authors:** Shaoxiong Zhai, Wenxu Dai, Jun Lin, Shaojian He, Bing Zhang, Lin Chen

**Affiliations:** Beijing Key Laboratory of Energy Safety and Clean Utilization, North China Electric Power University, Beijing 102206, China; ncepuzhaisx@163.com (S.Z.); dwx903005767@163.com (W.D.); jun.lin@ncepu.edu.cn (J.L.); 51101868@ncepu.edu.cn (B.Z.); chenlin@ncepu.edu.cn (L.C.)

**Keywords:** proton exchange membrane, sulfonated poly(ether ether ketone), sodium dodecyl benzene sulfonate, proton conductivity

## Abstract

It is of great importance to improve the proton conductivity of proton exchange membranes by easy-handling and cost-efficient approaches. In this work, we incorporated a commercially obtained surfactant, sodium dodecyl benzene sulfonate (SDBS), into sulfonated poly(ether ether ketone) (SPEEK) through solution casting to prepare SPEEK/SDBS membranes. When no more than 10 wt % SDBS was added, the SDBS was well dissolved into the SPEEK matrix, and the activation energy for the proton transfer in the SPEEK/SDBS membranes was greatly reduced, leading to significant enhancement of the membrane proton conductivity. Compared with the SPEEK control membrane, the SPEEK/SDBS membrane with 10 wt % SDBS showed a 78% increase in proton conductivity, up from 0.051 S cm^−1^ to 0.091 S cm^−1^, while the water uptake increased from 38% to 62%. Moreover, the SPEEK/SDBS membrane exhibited constant proton conductivity under a long-term water immersion test.

## 1. Introduction

Proton exchange membrane fuel cells (PEMFCs) have been considered a promising energy conversion device due to their advantages of high energy efficiency and low environmental impact [1]. Proton exchange membranes (PEMs) are the core components of PEMFCs, and proton conductivity is one of the most important properties for the PEMs [2,3]. Among the various PEMs, sulfonated poly(ether ether ketone) (SPEEK) has attracted great attention due to its good mechanical strength, high chemical stability, and low cost [4,5,6]. Generally, high proton conductivity can be achieved when SPEEK has a high degree of sulfonation (DS) [7,8], while the high DS also results in high water uptake, leading to poor dimensional stability.

A commonly used and effective way to improve the proton conductivity without much deterioration of the dimensional stability of SPEEK is to introduce inorganic fillers modified with sulfonate-containing components into the polymer matrix [9,10,11]. Such enhancements in proton conductivity are mostly due to the formation of additional proton transport pathways in the PEMs. For example, Liu et al. [12] utilized dopamine-initiated atom transfer radical polymerization to graft polystyrene sulfonic acid (PSSA) onto the surfaces of halloysite nanotubes (HNTs), and when adding 15 wt % of these modified HNTs, the proton conductivity of a SPEEK membrane at 25 °C increased by 54%. Jiang et al. [13] used sodium dodecyl benzene sulfonate (SDBS) adsorbed onto graphene oxide (GO) as a filler to enhance the proton conductivity of SPEEK, and the proton conductivity was increased from 39.5 mS cm^−1^ to 93.8 mS cm^−1^ by incorporating 8 wt % filler. However, the modification process of the inorganic filler is usually complicated and sometimes costly. Another effective method to improve the proton conductivity, especially under high temperature and low humidity, is to incorporate solid heteropolyacid (HPA) or liquid phosphoric acid (PA) into the PEMs [14,15]. Unfortunately, the easy leaching of HPA impedes the further application of HPA-filled composites as PEMs, so great efforts have been made to immobilize HPA [16,17,18,19,20], but these are still cost- and time-consuming.

Therefore, there is still a need to explore easy-handling and cost-efficient approaches to further improve the proton conductivity of SPEEK. SDBS is a kind of commonly used surfactant, with one end being hydrophilic and the other end being lyophilic, and the sulfonic acid groups of SDBS might facilitate the connecting of the hydrophilic phase of PEMs, resulting in enhanced proton conductivity. In this work, to create more proton transport pathways in SPEEK membranes, the commercially obtained surfactant SDBS was used to prepare SPEEK/SDBS membranes through solution casting. Molecular-level dispersion of a relatively high content of SDBS was obtained in the SPEEK matrix. The effects of the content of SDBS on the membrane behavior were extensively investigated in terms of ion exchange capacity (IEC), proton conductivity, water uptake, and mechanical properties.

## 2. Experimental

### 2.1. Materials

Poly(ether ether ketone) (PEEK) (Victrex 450PF) was purchased from Victrex (Lancashire, UK). SDBS (99%) and dimethylacetamide (DMAc) were provided by Alfa Aesar, Tianjian, China.

### 2.2. Sample Preparation

**Figure 1:**
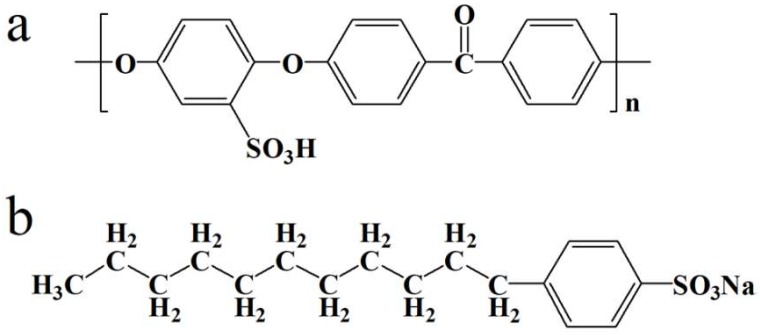
Structural formulae of (**a**) sulfonated poly(ether ether ketone) (SPEEK) and (**b**) sodium dodecyl benzene sulfonate (SDBS).

SPEEK was synthesized according to a method detailed in our previous work. To prepare SPEEK/SDBS membranes, both SPEEK and SDBS (with structural formulae shown in Figure 1a,b, respectively) were dissolved in DMAc, followed by stirring for 24 h at room temperature. After being degassed to remove air bubbles, the mixture was cast onto a flat glass substrate and dried at 80 °C for 24 h, and then further dried at 100 °C under vacuum for 18 h to thoroughly remove the DMAc. To replace the sodium ions of SDBS by H^+^, the as-prepared SPEEK/SDBS membranes with various SDBS contents were treated with 1 M H_2_SO_4_ and deionized (DI) water sequentially before use. For simplicity, the treated membranes were abbreviated as SPEEK/SDBS-*x* membranes, where *x* denotes the pre-introduced SDBS content (wt %).

### 2.3. Measurements

The sample cross section was obtained after breaking the membrane in liquid nitrogen followed by sputtering with gold. The morphology images were obtained via scanning electron microscopy (SEM) (SU8010, Hitachi Co. Ltd., Tokyo, Japan) at an accelerating voltage of 5 kV.

The IECs of the membranes were determined by an acid–base titration method according to the literature [21,22]. After immersing the dried membrane (~0.2 g) in 5.0 M NaCl solution (15 mL) for 24 h to release all H^+^, the amount of H^+^ was titrated with 0.01 M NaOH standard solution using phenolphthalein as an indicator. The IEC was calculated using the equation below:

(1) $$IEC=\frac{V\times c}{m},$$

where *V* and *c* are the volume and concentration of NaOH solution, respectively, and *m* is the weight of the dried membrane. The DS of the SPEEK was calculated to be 54% (for comparison, a SPEEK membrane with a DS of 73% was also prepared) from the following equation described in the literature [23]:

(2) $$DS=\frac{288\times IEC}{1000-102\times IEC}\times 100\%.$$

The dried membranes were immersed in deionized water at 25 °C for 6 h, and the wet membranes were obtained after the membranes were taken out and wiped with filter paper. The membrane water uptake was determined by the following equation:

(3) $$Water\;uptake=\frac{m_{w}-m_{d}}{m_{d}}\times 100\%,$$

where *m_d_* and *m_w_* are the masses of the dry and wet membranes, respectively.

Proton conductivity was calculated using the following equation:

(4) $$\sigma =\frac{L}{R\times w\times \delta },$$

where *L* is the spacing between the two electrodes; *w* and *δ* are the width and thickness of the samples, respectively; and *R* is the resistance measured in water via an AC impedance technique using an electrochemical workstation (Zennium Pro., Zahner, Germany).

According to ISO 1184-1983, the stress–strain curves of the membranes were recorded using an electrical tensile tester (AI-7000S1, Goodtechwill Testing Machines, Co. Ltd., China) at the speed of 2 mm min^−1^ under the relative humidity of 20%.

All the measurements were conducted at 25 °C unless mentioned otherwise.

## 3. Results and Discussion

### 3.1. SEM Observation of SPEEK/SDBS Membranes

**Figure 2:**
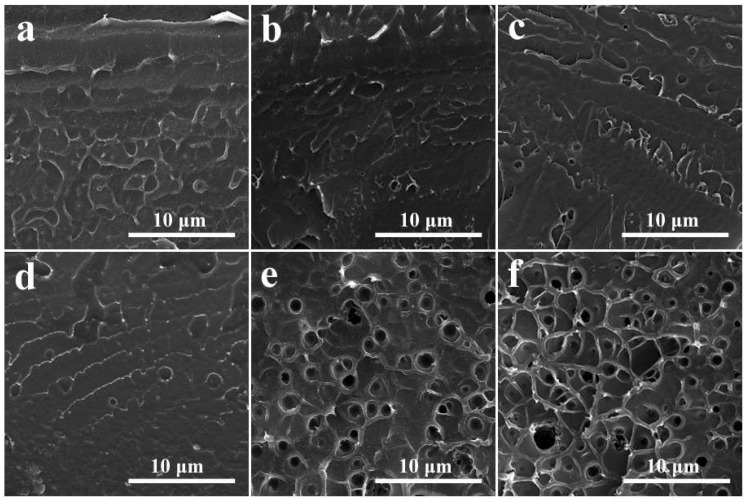
SEM images of (**a**) a SPEEK membrane and SPEEK/SDBS membranes with SDBS contents of (**b**) 2 wt %, (**c**) 4 wt %, (**d**) 10 wt %, (**e**) 15 wt %, and (**f**) 20 wt %.

SEM images of SPEEK and SPEEK/SDBS membranes with various SDBS contents are shown in Figure 2. As can be seen from Figure 2a, the freeze-fracture surface of the SPEEK membrane is relatively smooth without any visible voids. It can also be observed from Figure 2b–d that the surface morphologies of the SPEEK/SDBS membranes with SDBS contents of 2 wt %, 4 wt %, and 10 wt % are quite similar to that of the SPEEK membrane. Such similarity indicates that the SDBS could be well dissolved in the SPEEK matrix when no more than 10 wt % SDBS is incorporated; this should be due to the amphiphilic nature of both SPEEK and SDBS and the formation of ion clusters between the sulfonic acid groups of SPEEK and SDBS. The formation of ion clusters between the components containing sulfonic acid groups has been commonly observed in the literature [24,25]. For comparison, the freeze-fracture surfaces of SPEEK/SDBS membranes with SDBS contents of 15 wt % and 20 wt %, as shown in Figure 2e,f, respectively, are relatively rough, and some voids with the size of ~1 μm can be seen. When a large amount of SDBS (SDBS content higher than 10 wt %) is introduced, exceeding the saturation degree of SDBS in the SPEEK matrix, some SDBS should dissolve out from the SPEEK matrix, resulting in the formation of SDBS aggregates. However, these undissolved SDBS aggregates could be washed away during the membrane pre-treatment with 1 M H_2_SO_4_ and DI water, as mentioned in the experimental section; therefore, there exist some voids in the SPEEK/SDBS membranes with high SDBS content.

### 3.2. IECs of SPEEK/SDBS Membranes

**Figure 3:**
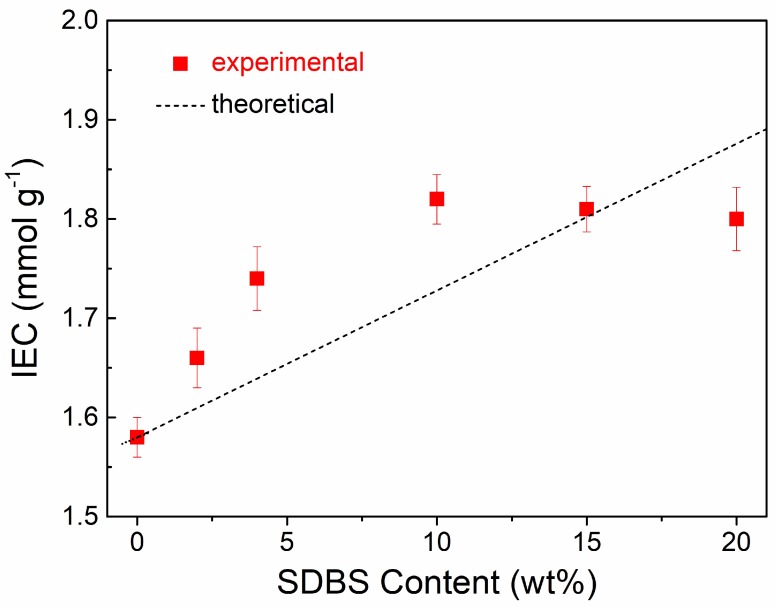
Ion exchange capacities (IECs) of SPEEK/SDBS membranes with various SDBS contents.

The IECs of the SPEEK/SDBS membranes with various SDBS contents are exhibited in Figure 3. The IECs of the SPEEK membrane and SDBS were determined to be 1.58 mmol g^−1^ and 3.06 mmol g^−1^, respectively. With increasing SDBS content, the IEC of the SPEEK/SDBS membranes first increases until it reaches a maximum of 1.82 mmol g^−1^ at 10 wt % SDBS content and then remains almost unchanged. Compared with SPEEK, SDBS exhibits a higher IEC, so the incorporation of SDBS into the SPEEK matrix could increase the IEC of the SPEEK/SDBS membranes. Additionally, we assume that the IECs of SPEEK/SDBS membranes linearly increase with increasing SDBS content; thus, the theoretical IECs of SPEEK/SDBS membranes should follow the dashed line shown in Figure 3, which is the connection between the IECs of SPEEK (SDBS content of 0 wt %) and SDBS (SDBS content of 100 wt %). However, it is found that the experimental IECs of SPEEK/SDBS membranes with no more than 10 wt % SDBS are much higher than the theoretical value, indicating that the protons for titrating are more dissociated than expected. Such a phenomenon should be attributed to the good dispersion of SDBS in the SPEEK matrix and the promotion of proton dissociation through ion clusters between SPEEK and SDBS when no more than 10 wt % SDBS is added. In comparison, when the SDBS content is higher than 10 wt % in the membranes, the leakage of undissolved SDBS occurs, as illustrated in the SEM observation mentioned above, which should not lead to an increase in the actual SDBS content in the membrane with further SDBS addition. As a result, the SPEEK/SDBS membranes with SDBS contents of 15 wt % and 20 wt % show almost the same IEC as the membrane with 10 wt % SDBS.

### 3.3. Proton Conductivity and Water Uptake of SPEEK/SDBS Membranes

**Figure 4:**
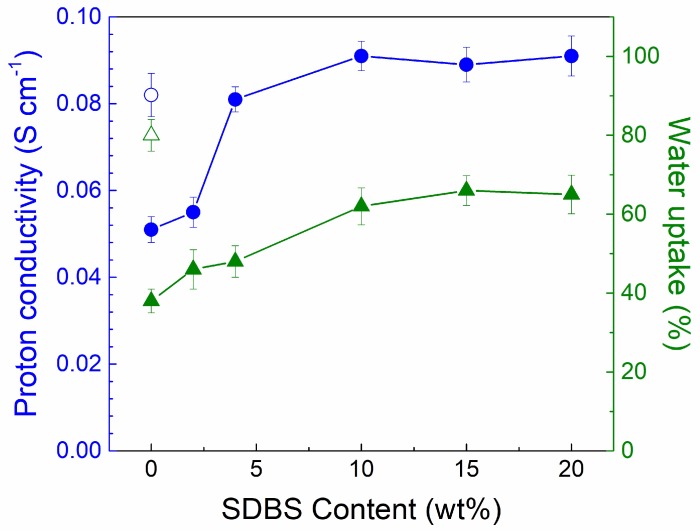
Proton conductivity and water uptake of SPEEK/SDBS membranes (solid symbols) with various SDBS contents and a SPEEK membrane with a degree of sulfonation (DS) of 73% (open symbols)

The proton conductivity and water uptake of the SPEEK/SDBS membranes with various SDBS contents are exhibited in Figure 4. For the membrane water uptake, a gradual increase with SDBS is expected because the hygroscopic SDBS tends to absorb more water than does SPEEK, and the voids created by the leakage of SDBS are responsible for the slightly increased water uptake at high SDBS content. Moreover, with increasing SDBS content, the proton conductivity of the SPEEK/SDBS membranes greatly increases at first and then levels off, which is consistent with the change in the membrane IEC. Such an enhancement in the membrane proton conductivity with low SDBS addition should be due to two main factors. Firstly, SDBS has a higher IEC and thus can dissociate more protons than SPEEK, so the introduction of SDBS can create more continuous proton transfer channels in the membrane. Secondly, the formation of ion clusters between the sulfonic acid groups of SPEEK and SDBS could result in stronger dissociation of protons, further facilitating the hopping of protons. However, the actual SDBS content in the membrane does not increase with further increasing SDBS content above 10 wt %, thus leading to unchanged proton conductivity. It should be noted that the formation of ion clusters between the sulfonic acid groups of SPEEK and SDBS would not lead to an additional increase in water uptake. As a result, the SPEEK/SDBS membrane with 10 wt % SDBS exhibits much higher proton conductivity than the SPEEK membrane, with the proton conductivity rising from 0.051 S cm^−1^ to 0.091 S cm^−1^, while the water uptake increases from 38% to 62%. For comparison, the SPEEK membrane with a DS of 73% only achieves the proton conductivity of 0.082 S cm^−1^ with relatively high water uptake (80%). Although the enhancement in proton conductivity in our work was not as high as that of the previous reported SPEEK membrane using SDBS-adsorbed graphene oxide (GO) as a filler (the proton conductivity rising from 39.5 mS cm^−1^ to 93.8 mS cm^−1^ by using 8 wt % filler) [13], to improve the proton conductivity by directly incorporating SDBS into SPEEK is a more cost-efficient approach and is easier to handle.

### 3.4. Activation Energy for Proton Conduction in SPEEK/SDBS Membranes

**Figure 5:**
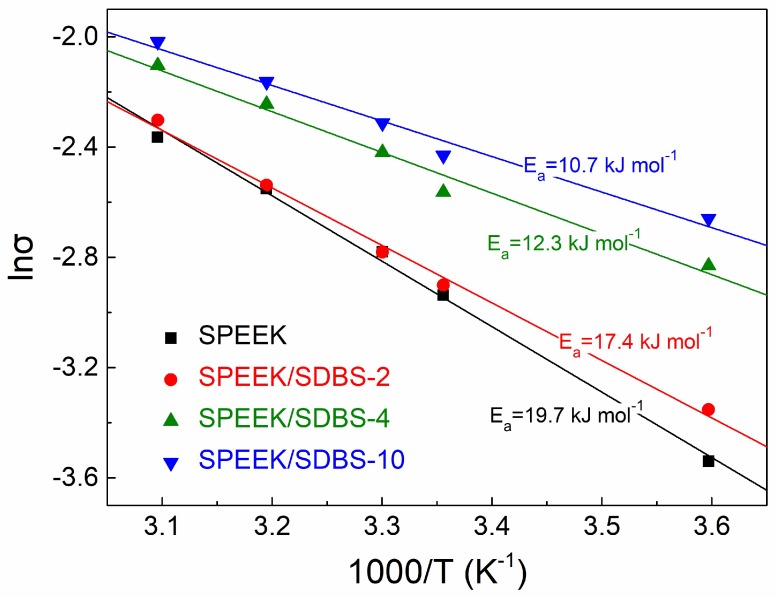
Arrhenius plots of proton conductivity for a SPEEK membrane and SPEEK/SDBS membranes with SDBS contents of 2 wt %, 4 wt %, and 10 wt % as a function of temperature.

The activation energy for proton conduction (*E_a_*) reveals the minimum energy required for proton transport from one free site to another, and a low *E_a_* indicates lower energy loss caused by ionic resistance of the membrane, which is beneficial for the energy utilization of PEMFCs [13]. Figure 5 shows the Arrhenius plots of proton conductivity (*σ*) for SPEEK and SPEEK/SDBS membranes with SDBS contents of 2 wt %, 4 wt %, and 10 wt %, and *E_a_* (kJ mol^−1^) can be derived from the linear fitting of the plots using the following equation [26]:

(5) $$\ln\sigma =\ln A-\frac{E_{a}}{RT},$$

where *A* is the pre-exponential factor, *R* is the ideal gas constant (8.314 J K^−1^ mol^−1^), and *T* is the temperature (K).

**Figure 6:**
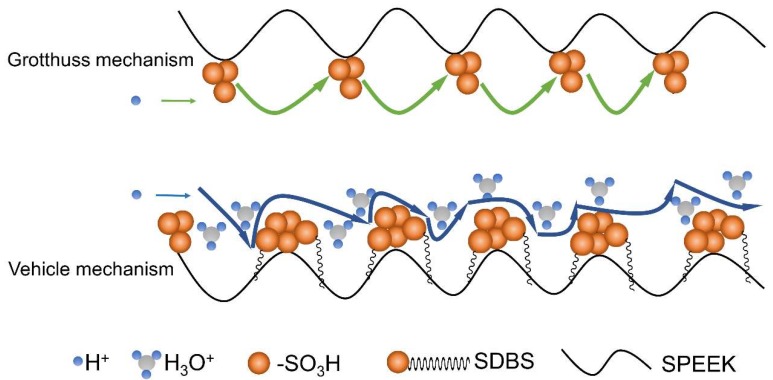
Schematic diagram of proton transport mechanisms in the SPEEK/SDBS membranes.

As is well known, there are two main mechanisms [27,28,29,30]—Grotthuss and vehicle mechanisms—responsible for the proton transport in hydrated membranes. For the Grotthuss mechanism, protons jump between adjacent sulfonic acid groups or water molecules to achieve proton transfer, while for the vehicle mechanism, the protons are transferred by −SO_3_H and some complexes such as H_3_O^+^ and H_5_O_2_^+^. The calculated *E_a_* for a SPEEK membrane is 19.7 kJ mol^−1^, which lies within the reported *E_a_* range for the Grotthuss mechanism (14.0~40.0 kJ mol^−1^) [31], indicating that the proton conduction in the SPEEK membrane is mainly dominated by the Grotthuss mechanism. For comparison, the calculated *E_a_* values for the SPEEK/SDBS-2, SPEEK/SDBS-4, and SPEEK/SDBS-10 membranes are 17.4 kJ mol^−1^, 12.3 kJ mol^−1^, and 10.7 kJ mol^−1^, respectively. These are lower than that of the SPEEK membrane and decrease with increasing SDBS content until the SDBS content reaches 10 wt %. When SDBS is added into the membrane, the hydrophilic sulfonic acid groups in SDBS contribute to the water absorption of the membranes, and more water molecules might help to construct a proton-conducting pathway and improve the proton transfer ability via the vehicle mechanism; therefore, much lower *E_a_* values are achieved by the SPEEK/SDBS membranes. The proton transfer mechanisms, including the Grotthuss and vehicle mechanisms, in the SPEEK/SDBS membranes are shown in Figure 6.

### 3.5. Stability of SPEEK Nanocomposite Membranes

**Figure 7:**
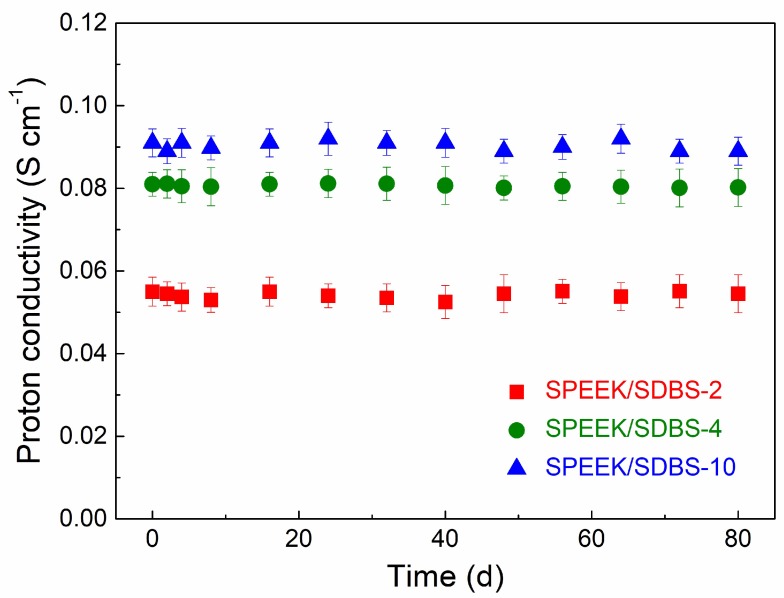
Proton conductivity of the SPEEK/SDBS-10 membrane as a function of immersion time in deionized (DI) water.

Generally, the leakage of water-soluble low-molecular-weight compounds in PEMs is extremely serious, so it is quite necessary to evaluate the stability of SDBS in the SPEEK membrane. The SPEEK/SDBS-2, SPEEK/SDBS-4, and SPEEK/SDBS-10 membranes were immersed in water that flowed continuously, and proton conductivities were tested at various times to check the stability of the membranes. As shown in Figure 7, the proton conductivities of the three SPEEK/SDBS membranes remained almost constant after 80 days, indicating that SDBS is quite stable in the membranes without leaking. Additionally, the weight of the membranes remains constant during the test, also illustrating that the SDBS could not have leaked out. These results can be explained as follows: SDBS is an amphiphilic molecule with one end being hydrophilic and the other end being lyophilic. The relatively long alkyl chain of SDBS can be entangled with hydrophobic-phase macromolecules in SPEEK, while the sulfonic acid groups of SDBS can form ionic clusters with the sulfonic acid groups in SPEEK. Therefore, the migration of SDBS would encounter large resistance in either the hydrophilic or the hydrophobic phase. The SEM images also confirm that SDBS is well embedded in SPEEK when the loading of SDBS does not exceed 10 wt %.

### 3.6. Mechanical Properties of SPEEK/SDBS Membranes

**Figure 8:**
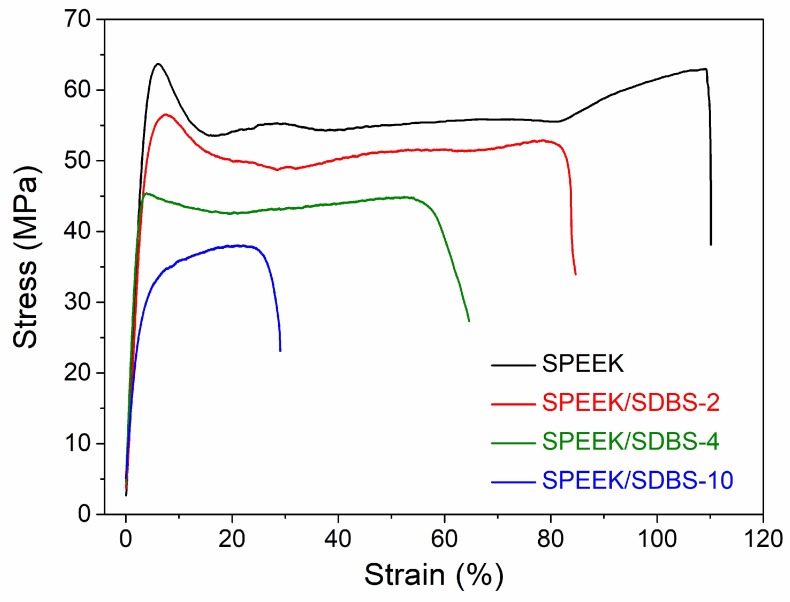
Stress–strain curves of a SPEEK membrane and SPEEK/SDBS membranes with SDBS contents of 2 wt %, 4 wt %, and 10 wt %.

Tensile tests were performed to investigate the effect of SDBS on the mechanical properties of SPEEK/SDBS membranes, and the results are shown in Table 1. The stress–strain curves are presented in Figure 8. Compared to the SPEEK membrane, the SPEEK/SDBS membranes demonstrate lower elastic moduli, mainly due to the incorporation of soft organic SDBS acting as a plasticizer. The yield strength, tensile strength, and elongation at break of the SPEEK/SDBS membranes are also lower than those of the SPEEK membrane because the addition of SDBS may increase the space between the polymer chains, and the molecular inter-atomic force is thus damaged at some level. Nevertheless, the SPEEK/SDBS membranes still possess sufficiently high mechanical properties to be applied in fuel cells.

## 4. Conclusions

In this work, a commercially available surfactant, SDBS, was incorporated into SPEEK through solution casting to prepare SPEEK/SDBS membranes. As illustrated by SEM observations, SDBS was well dissolved into the SPEEK matrix when no more than 10 wt % SDBS was added, and leakage of SDBS occurred in the SPEEK/SDBS with SDBS content higher than 10%. By incorporating SDBS, the activation energy for the proton transfer in the membranes was greatly reduced due to the formation of more continuous proton transfer channels. Compared with the SPEEK control membrane, the SPEEK/SDBS membranes showed significantly enhanced proton conductivity with relatively low water uptake because the formation of ion clusters between the sulfonic acid groups of SPEEK and SDBS could promote proton dissociation without leading to an additional increase in water uptake. The proton conductivity of the SPEEK/SDBS membrane was constant during long-term water immersion tests.

## Figures and Tables

**Table 1:** Mechanical properties of SPEEK/SDBS membranes.

Performance	Elastic Modulus (GPa)	Yield Strength (MPa)	Tensile Strength (MPa)	Elongation at Break (%)
SPEEK	1.72	63.7	63.0	110
SPEEK/SDBS-2	1.70	56.6	52.8	85
SPEEK/SDBS-4	1.42	45.4	44.9	58
SPEEK/SDBS-10	0.92	-	37.9	29
